# Barriers and facilitators to mood and confidence in pregnancy and early parenthood during COVID-19 in the UK: mixed-methods synthesis survey

**DOI:** 10.1192/bjo.2021.925

**Published:** 2021-06-01

**Authors:** Alejandra Perez, Elena Panagiotopoulou, Peter Curtis, Ruth Roberts

**Affiliations:** Education & Training Division, Academic & Research Department, Anna Freud National Centre for Children and Families, UK; and Research Department of Clinical, Educational & Health Psychology, University College London, UK; Education & Training Division, Academic & Research Department, Anna Freud National Centre for Children and Families, UK; and Research Department of Clinical, Educational & Health Psychology, University College London, UK; Department for Education, UK; Education & Training Division, Academic & Research Department, Anna Freud National Centre for Children and Families, UK; and Research Department of Clinical, Educational & Health Psychology, University College London, UK

**Keywords:** Parental well-being, parenting, infancy, pregnancy, COVID-19

## Abstract

**Background:**

Parental well-being during pregnancy and early parenthood is critical for child development. Environmental stressors can significantly challenge parental well-being.

**Aims:**

To investigate how COVID-19 and associated restrictions influence mood and parenting confidence of expectant parents and those in early parenthood, identifying barriers and facilitators.

**Method:**

We used a cross-sectional online survey to collect data from 590 expectant parents and parents of infants (564 women) during the most restrictive phase of lockdown in the UK. We included a mixture of forced-choice and open-ended questions pertaining to mood, perceived social support, media use, online interactions and parenting expectations. Quantitative data were analysed with multiple linear regression and proportional odds models; an inductive thematic analysis was used for qualitative data. Quantitative and qualitative data were qualitatively synthesised.

**Results:**

Since COVID-19, expectant parents and parents of new-borns reported a decrease in mood and parenting confidence. Barriers included practical difficulties (finding essentials, reliable health information), social difficulties (loss of physical contact, decreased support) and uncertainty during pregnancy. Facilitators included support from others and, for first-time parents, loss of child care resulting in greater parenting confidence. Although online resources and communication were not preferable to face-to-face interactions, technology was a helpful tool for communicating, getting support, and finding essentials and information during lockdown.

**Conclusions:**

By mid-May 2020, mood and parenting confidence among expectant and parents of new-borns in the UK were significantly reduced. Consideration of barriers and facilitators in healthcare and psychological support provided is likely important for promoting parental mental health and healthy parent–child relationships.

The response to prevent and reduce infection of COVID-19 in the UK involved several changes to people's lives and healthcare settings. From 23 March to 11 May 2020, UK Government guidance was for people to stay at home, except for very limited purposes (e.g. shopping for basic necessities, one form of exercise a day, medical need, etc.), certain businesses and venues were closed and gatherings of more than two people in public were stopped.^1^ Pregnant women were defined as a vulnerable group as a precaution by the Chief Medical Officer on 16 March 2020, and were advised to reduce social contact through social distancing measures, with increased stringent measures from 28 weeks of pregnancy onward.^2^ Access to maternity services was limited; for example, partners were not able to attend antenatal or scanning appointments, only one birthing partner was able to be present and only for established labour and birth, partners were not permitted on the postnatal ward,^2^ restrictions were placed on births at home and in freestanding midwifery units in certain areas,^3^ there was a decrease in antenatal and postnatal appointments,^4^ and some appointments were conducted via telephone or video instead of face to face.^2^ Community services, such as breastfeeding support and some health-visiting appointments, were moved remotely as were antenatal and postnatal groups.^4^ These guidelines are continuously updated and under review. The time taken to implement modifications in maternity services and the extent of restrictions has varied across the UK.^5^

Parental well-being is an important factor for parenting, parent–infant relationships and healthy child development. A large body of literature has accumulated strong evidence directly linking parental depression with poor parenting practices, such as hostile or disengaged parenting^6^ and poorer mother–infant quality interactions,^7^ whereas maternal and paternal depressive symptoms and parenting stress of both parents can be risk factors for the socio-emotional, behavioural and functional development of children.^8–10^ In addition to parents’ mood, confidence in parenting has also been found to influence parental care and child development, with a higher parental self-efficacy influencing capacity to carry out positive parenting practices^11,12^ and child adjustment.^13,14^ Importantly, parental mental health and confidence can be challenged significantly in the face of environmental stressors, such as a health-related threat. Parents’ perception of child vulnerability has been found to be associated with postnatal depression and parental stress, which, in turn, affect parent–child relationships,^15,16^ as well as perceptions of themselves as less competent parents.^17^ To this end, it seems vital to understand the impact of COVID-19 and associated restrictions on the well-being of expectant parents and parents of new-borns, as well as their perception of themselves as parents. This will help inform where attention and priority should be given to address the needs of this population. The aim of this study was, therefore, to investigate how COVID-19 and associated restrictions influence mood and parenting confidence of expectant parents and those in early parenthood, identifying barriers and facilitators.

## Method

### Participants

Online recruitment included 590 participants (564 women). The inclusion criteria were that participants must be currently pregnant, have a pregnant partner or a baby <6 months old; living in the UK and >18 years of age. Before their participation, all participants were provided with an information sheet and written informed consent was obtained from all participants.

The study was approved by the Ethics Committee of the Research Department of Clinical, Educational and Health Psychology, University College London (project identifier 7683/003).

### Design and materials

This cross-sectional survey was conducted on UCL Qualtrics (Qualtrics LLC, London, UK; see https://login.qualtrics.com) and included questions pertaining to demographic information, mood and general well-being, stress events, perceived support from others, media use, experience of online interactions, sudden loss of physical contact, expectations of parenting, and hardest and most helpful experiences during the crisis. Some of the questions had forced-choice answers on a yes/no basis or a Likert rating scale. The survey also collected qualitative data through three open-ended questions, to gain more in-depth information and put the answers into context. The questions were developed by three of the authors, who have research and clinical background in child mental health and parenting, and piloting on three individuals was undertaken to ensure that questions were brief, relevant, unambiguous and were not leading participants to answer in a particular way. The study used a convergent synthesis design, whereby qualitative and quantitative data were collected and analysed in parallel, before being qualitatively synthesised. For a full version of the survey, please refer to Supplementary Appendix 1 available at https://doi.org/10.1192/bjo.2021.925.

### Procedure

Data collection took place between 24 April 2020 and 13 May 2020, during the first and most severe phase of the nationwide lockdown in the UK. A recruitment flyer, including information about the study, inclusion criteria and the link to the survey, was posted/reposted on social media, as well as on the MQ Mental health research website and a civil service message board. The survey was anonymous and took no more than 10 min to complete. After submitting the answers, a list of links to resources for confidential support and advice was provided.

### Data analysis

#### Quantitative

Variables of interest were grouped into four themes: support, physical contact, media use and stress events. Models for the support theme included the following variables: current support from partners, friends and family; and the change in support from partners, friends and family since COVID-19. Change in support is calculated by scoring the ordered categories in the current and prior support variables from 1 to 4, with 1 indicating no support and 4 indicating a high level of support, and deducting prior from current support, giving a number between −3 and 3, with negative values indicating a decrease in support. Models for the physical contact theme included the following variables: worries about having physical contact with others in the household, avoiding contact with others in the household and missing physical contact more generally. Models for the use of media theme included the following variables: preference of connecting with others online, frequency of looking at social media, frequency of looking at news, frequency of searching for reliable health information and finding a reliable source of health information. Models for the stress events theme included a cumulative number of stress events experienced (out of a total of 11) and the four most frequently reported stress events: having to isolate from friends/family members; having to attend hospital/general practitioner appointments on their own; having difficulty accessing medicine, groceries or other essential items; and losing access to child care.

Two outcomes were examined: change in mood since COVID-19 and impact of COVID-19 on confidence in parenting. Change in mood is calculated by scoring the ordered categories in the current and prior mood variables from 1 to 5, with 1 indicating very sad and 5 indicating very happy, and deducting prior from current mood, giving a number between −4 and 4, with negative values indicating a decrease in mood. Mood before COVID-19 was included as a covariate when modelling change in mood, as it is correlated and so needs to be controlled for. Change in mood was examined with multiple linear regression models.

Parenting confidence was measured by asking participants how they felt as an expectant parent or parent since COVID-19, and were given three options: more confident, same as before or less confident. Parenting confidence was examined with proportional odds models. Participant characteristics, including gender, age, expectant/new parent status, previous parent experience, work situation, income, accommodation and location in the UK, were included as covariates for variable selection. Full details of the analysis are reported in Supplementary Appendix 2.

In the total sample, 26 participants did not identify as female. Because of the potential effects of gender on responses, analyses were duplicated with female participants only. No conclusions changed in any of the female-only analyses. Therefore, the analyses presented in this paper included all participants (unless specified otherwise, e.g. pregnant participants only).

#### Qualitative

An inductive thematic analysis was conducted. The researchers initially familiarised themselves with the data-set and coded 35 responses each (two out of three open-ended questions). They discussed their coding and organised lists of codes into a coding framework, with inclusion and exclusion criteria for each code. The same two independent raters recoded 11.8% of the total sample (70 responses; half from the pregnant group and half from the parent group) for two out of three questions, using this coding framework. High interrater reliability was established (*κ* > 0.85), so the raters divided and coded the remaining data separately. Ambiguous instances were resolved through discussion, and the two raters developed and refined themes together.

Using the original coding framework, the same two independent raters separately analysed qualitative responses provided by men only, to determine if there were any different themes particular to male participants. It was confirmed that the themes developed were representative of both women and men, so the qualitative analyses presented in the paper included all participants.

### Data synthesis

Quantitative and qualitative data were qualitatively synthesised to help understand the directionality of findings, generating a new set themes for barriers and facilitators, in line with best practice when aggregating data from different types of research.^18^

Two authors conducted the quantitative analyses and two different authors conducted the qualitative analyses, to avoid biases. All authors were involved in data synthesis.

To identify barriers and facilitators to participants’ change in mood, as well as parenting confidence, the quantitative and qualitative data were synthesised. The quantitative analysis revealed factors that were associated with a decrease in mood and confidence since COVID-19. However, the associations could be reversed, meaning that it is not possible to know whether each of these factors is a barrier or (if reversed) a facilitator. For this reason, the qualitative data were used to understand more about potential directionality of the associations. Further details are described in the Results section.

## Results

### Quantitative analysis

Full participant characteristics are displayed in Table 1. Since the start of the COVID-19 pandemic, 68% of respondents reported a drop in mood and 25% of respondents reported feeling less confident. Table 2 reports on additional contextual study highlights pertaining to physical contact; worries about own and baby's health; use of technology and information; stressful life events and support from partner, family and friends.

**Table 1:** Participant characteristics

	*n* (% of total sample)		
	Expectant parent	New parent	Total
Gender			
Female	355 (60.2)	209 (35.4)	564 (95.6)
Male	10 (1.7)	15 (2.5)	25 (4.2)
Specify	0 (0.0)	1 (0.2)	1 (0.2)
Age, years			
18–24	31 (5.3)	7 (1.2)	38 (6.4)
25–34	235 (39.8)	143 (24.2)	378 (64.1)
35–44	99 (16.8)	75 (12.7)	174 (29.5)
≥45^a^	0 (0.0)	0 (0.0)	0 (0.0)
Previous parent experience			
First	161 (27.3)	142 (24.1)	303 (51.4)
Not first	204 (34.6)	83 (14.1)	287 (48.6)
Work situation			
Working in the NHS or as a care worker	63 (10.7)	23 (3.9)	86 (14.6)
Working in public facing role (not NHS or care worker)	53 (9.0)	31 (5.3)	84 (14.2)
Not working with public, NHS or care worker	249 (42.2)	171 (29.0)	420 (71.2)
Income			
Not supplied	7 (1.2)	2 (0.3)	9 (1.5)
Up to £20 000	42 (7.1)	14 (2.4)	56 (9.5)
£21 000–£40 000	71 (12.0)	39 (6.6)	110 (18.6)
£41 000–£60 000	92 (15.6)	52 (8.8)	144 (24.4)
£61 000–£80 000	72 (12.2)	47 (8.0)	119 (20.2)
£81 000–£100 000	34 (5.8)	35 (5.9)	69 (11.7)
≥£100 000	47 (8.0)	36 (6.1)	83 (14.1)
Accommodation			
Not supplied	2 (0.3)	1 (0.2)	3 (0.5)
Flat with no view	17 (2.9)	8 (1.4)	25 (4.2)
Flat with view/balcony	22 (3.7)	19 (3.2)	41 (7.0)
Flat with garden/terrace/outside space	28 (4.8)	21 (3.6)	49 (8.3)
House with garden/terrace/outside space	296 (50.2)	176 (29.8)	472 (80.0)
Location in the UK			
Not supplied/indeterminate^b^	12 (2.0)	16 (2.7)	28 (4.8)
North-East England	13 (2.2)	4 (0.7)	17 (2.9)
North-West England	22 (3.7)	11 (1.9)	33 (5.6)
South-East England	65 (11.0)	26 (4.4)	91 (15.4)
South-West England	27 (4.6)	34 (5.8)	61 (10.3)
East Midlands	22 (3.7)	6 (1.0)	28 (4.8)
East of England	40 (6.8)	17 (2.9)	57 (9.7)
West Midlands	19 (3.2)	8 (1.4)	27 (4.6)
Yorkshire and The Humber	28 (4.7)	19 (3.2)	47 (8.0)
London	81 (13.7)	69 (11.7)	150 (25.4)
Scotland	22 (3.7)	9 (1.5)	31 (5.3)
Wales	7 (1.2)	5 (0.8)	12 (2.0)
Northern Ireland	7 (1.2)	1 (0.2)	8 (1.4)

Because of a technical issue with data collection, ethnicity was not obtained for the majority of participants. This error was corrected as soon as it was identified. In the sample that followed, 47 participants were White, 2 participants were of mixed ethnicity and 1 participant was Asian. NHS, National Health Service.

a. The option of ≥45 years was provided but no participant selected this option.

b. Location information was not given by 12 participants, and location could not be determined from the information provided by 14 participants (e.g. ‘rural village’; it could not be determined if ‘Richmond’ was in London or Yorkshire). Two participants indicated that they were not living in the UK and were removed from all analyses.

**Table 2:** Study highlights

% of total respondents (*n/N*)	Descriptive Findings
68.0 (401/590)	Reported a drop in mood since COVID-19
25.2 (147/584)	Reported feeling less confident since COVID-19
53.5 (312/583)	Reported that the experience of COVID-19 will influence their (future) parenting
74.8 (439/587)	Reported missing physical contact with others ‘a lot’ or ‘a great deal’ since COVID-19
87.6 (517/590)	Reported feeling more worried about going to hospital/medical appointments or home visit since COVID-19
73.9 (434/587)	Reported feeling more worried about baby's health since COVID-19
69.3 (409/590)	Reported feeling more worried about their own health since COVID-19
84.1 (481/572)	Found connecting with others online less preferable than face-to-face since COVID-19
75.0 (439/585)	Reported looking at social media more often since COVID-19
69.5 (408/587)	Reported looking at the news more often since COVID-19
64.9 (377/581)	Reported searching for reliable health information more often since COVID-19
61.2 (334/546)	Indicated that they had found a reliable source of health information (majority reported NHS)
91.2 (538/590)	Reported isolating from friends/family members^a^
78.1 (461/590)	Reported attending hospital/general practitioner appointments on their own^a^
50.5 (298/590)	Reported having difficulty accessing medicine, groceries or other essential items^a^
33.4 (197/590)	Reported losing access to child care^a^
72.9 (422/579)	Reported feeling highly supported by partner since COVID-19
11.6 (67/577)	Reported a reduction in partner support since COVID-19
35.4 (208/588)	Reported feeling highly supported by family since COVID-19
43.5 (254/584)	Reported a reduction in family support since COVID-19
21.6 (126/584)	Reported feeling highly supported by friends since COVID-19
49.6 (287/579)	Reported a reduction in support from friends since COVID-19

NHS, National Health Service.

a. Four most commonly reported stress events.

#### Change in mood since COVID-19

A decrease in mood was associated with an increase in missing physical contact more generally and an increase in avoiding physical contact with others in the household (*F*(6, 576) = 55.94, *P* < 0.0001, *R^2^* = 0.37); not finding reliable health information (*F*(5, 533) = 57.92, *P* < 0.0001, *R^2^* = 0.35); and difficulty in accessing medicine, groceries or other essential items (*F*(4, 585) = 83.79, *P* < 0.0001, *R^2^* = 0.36). In models examining physical contact and support, being pregnant was associated with a decrease in mood. An increase in mood was associated with higher levels of current support from partner and friends, and an increase in family support (*F*(8, 553) = 47.00, *P* < 0.0001, *R^2^* = 0.40). Model summaries appear in Supplementary Appendix 3.

#### Change in confidence since COVID-19

A decrease in confidence as a parent was associated with an increase in missing physical contact more generally (odds ratio 0.83, 95% CI 0.70–0.98); an increase in searching for reliable health information (odds ratio 0.69, 95% CI 0.50–0.93); and difficulty in accessing medicine, groceries or other essential items (odds ratio 0.46, 95% CI 0.32–0.64). An increase in confidence as a parent was associated with loss of access to child care (odds ratio 1.52, 95% CI 1.05–2.21) and increase in family support (odds ratio 1.33, 95% CI 1.03–1.73).

### Qualitative analysis

Three thematic analyses were conducted for the following open-ended questions: (a) ‘What has been the hardest part of being pregnant/parent of a new baby during COVID-19?’; (b) ‘What have you found to be most helpful while being pregnant/parent of a new baby during COVID-19?’; and (c) ‘Do you think this experience of COVID-19 will influence your future parenting/parenting? No/Yes. Please tell us below.’ Analyses revealed seven themes for question (a), five themes for question (b) and six themes for question (c) (see Tables 3–5).

**Table 3:** Themes, explanations and submission examples for what was ‘hardest’, in order of frequency

Theme	Explanation and examples of submissions assigned to each theme
Decreased support	From partner (not being allowed at appointments and full labour), family and friends, healthcare and antenatal groups, and for child care
Pregnant group	From partner: ‘I have my 12-week scan next week and my husband can't come with me (due to hospitals COVID instructions) and this made me very anxious. If something is not right at the scan, I won't have anyone there with me to support me. This has made me really anxious and since I'm working from home and am deprived of normal social interactions and distractions, it has played on my mind constantly.’ From healthcare: ‘Fewer antenatal appointments, 36-week scan was cancelled - so worried about quality of care and whether issues with my pregnancy may be being missed.’ For child care: ‘We live far away from family and have no one to look after our 2 year old when I have the new baby. The original plan was for family to stay and support us. Also having to look after my toddler whilst working remotely as a teacher whilst my husband also works from home has been extremely tiring and I have felt a lot of guilt about the amount of TV she has watched…’
Parent group	From family: ‘Not having the extra support of having family here to help with our toddler and to be able to come and see the baby.’ From healthcare: ‘Lack of midwife/medical support, to be able to ask questions when you aren't certain of things. Baby having a medical condition, but not having the medical support. Having specialist appointments cancelled without being told. Having to chase for appointments to be made, where they would usually be made automatically. Not being able to attend clinics for reassurance.’ From postnatal groups: ‘Not being able to meet up with other parents and go to baby groups - a lot of anecdotal knowledge is shared in these situations, which I'm definitely missing.’
Loss	Of seeing family and friends, missing out on markers of pregnancy and early parenthood, birth choice, enjoying this time and baby missing out on normal experience
Pregnant group	Missing out on markers: ‘Being alone, missing out on first time mum things, like seeing friends, enjoying last weeks of pregnancy, no baby shower/celebrations, not getting to ‘show off’ bump. Have some family members that I haven't seen whilst being visibly pregnant, going out to choose baby items.’ Birth choice: ‘Have lost my midwife and am no longer able to have a home birth - now will be in hospital.’ Of seeing friends and family: ‘It's really hard to get excited about pregnancy when all the special moments that you want to share with your friends and partner can't really happen. I imagined holding his hand looking at our first scan together. Doing it alone just feels lonely and sad.’
Parent group	Of seeing friends and family: ‘Not seeing family - neither set of grandparents have met their grandchild.’
Worry	About contracting COVID-19 (and its effect on pregnancy and baby), birth in these circumstances, the impact of social isolation (on baby's development and own mental health), financial worries and family's health
Pregnant group	About contracting COVID-19: ‘Very lonely, rather than enjoying your pregnancy you're worried the entire time because the thought of getting it takes over, especially when there isn't enough research to show how it affects pregnant women and their babies.’
Parent group	About contracting COVID-19: ‘Worrying about what will happen to our children if my husband and I get COVID-19 and will need to be in hospital at the same time. We have no immediate family nearby and who is going to take in a couple of children who might carry coronavirus? This thought fills me with dread.’ Impact of social isolation on baby's development: ‘Lack of social interactions with family, friends and wider community, which, besides being important for my baby's social development, is affecting my mood (and my mood might affect my motherhood).’ Financial worries: ‘My partner has been furloughed and I am on maternity pay so our income has decreased dramatically and we are struggling.’
Having to be at home	Feeling lonely, isolated and stuck with no change, and not able to see others, exercise or work, and finding being exclusively with baby ‘intense’
Pregnant group	‘Not being able to go places in the car that lift my spirits, such as the beach, for lunches out, etc as I can't walk far from my house being so heavily pregnant with sciatica.’
Parent group	‘Socially isolated from everyone and therefore feeling overwhelmed with the responsibility of looking after my baby.’
Uncertainty during pregnancy	Not knowing the risks on pregnancy and baby, on what life will be like and on birth
Pregnant group	‘Not knowing what's happening, will I have to give birth alone? Will we be ok?’
Parent group	‘Not seeing my parents and the uncertainty about when we will be seeing them (they live abroad) when they will finally meet their granddaughter.’
Practical difficulties	Not able to go to shops and find things online
Pregnant group	‘Difficulty getting baby supplies, organising what will happen when I go into labour.’
Parent group	‘Initially buying formula as everyone went crazy bulk buying and there was none in the shops.’
Unreliable information	About COVID-19, healthcare arrangements, pregnancy and postnatal care, and birthing
Pregnant group	‘Conflicting information and news reporting is extremely confusing and health professionals are often only able to cite the basic government advice which doesn't feel substantial.’
Parent group	‘It was difficult finding good information at first. For example, we weren't sure whether to go ahead with her 16 week vaccinations and I couldn't find any information online. However, that has now changed.’

**Table 4:** Themes, explanations and submission examples for what was ‘most helpful’, in order of frequency

Theme	Examples of submissions assigned to each theme
Support from others	Partner, family and friends, healthcare professionals, antenatal and postnatal groups, employer and therapy
Pregnant group	From partner: ‘Talking to my partner.’ From healthcare: ‘My partner and all the telephone appointments from doctors/midwives. Never felt let down by them.’
Parent group	From partner: ‘Having my partner at home more during the day to help with the baby.’ From healthcare: ‘Having healthcare professionals still engaging and checking we're ok via phone calls.’
More time	‘As a family’, with partner, with baby, ‘me-time’ inside and outside the house, and for planning arrival of baby
Pregnant group	‘Being at home and having time to prepare for baby.’
Parent group	‘More attention on baby and being able to get into a good routine with no interruptions. Also dad not missing out on any developmental stages as working from home.’
Technology	Availability of online resources, for live communication, social media, apps, shopping and online information
Pregnant group	Availability of online resources: ‘How many things have gone online.’ Online information: ‘RCOG [Royal College of Obstetricians and Gynaecologists] guidelines are clear and have evidence written in a clear way to support, have helped reassure me and I used these to insist on a risk assessment for work.’ Social media: ‘Technology and Wi-Fi! Being able to send updates. Being able to create a group to network with local mums to be.’
Parent group	Live communication: ‘Access to digital technology (Skype, Zoom, WhatsApp, etc.) that have enabled me to keep in touch with family, particularly video calls so that they can see how our baby is growing even if they can't see her in person.’
No pressure	Feeling less stressed about not having to commute, work, meet people and rush things
Pregnant group	‘Not feeling pressured to go outside/do things.’
Parent group	‘Not having the pressure to be anywhere or see people is fairly helpful when you've not slept, feel exhausted and can't manage to get dressed!’
Nothing	‘Nothing’ written down
Pregnant group	‘Nothing.’
Parent group	‘Nothing.’

**Table 5:** Themes, explanations and submission examples for ‘influence on future parenting/parenting’, in order of frequency

Theme	Examples of submissions assigned to each theme
Hygiene and restrictions of socialising	Worries directly related to virus: restricting own and child's social interactions; being more hygienic; and feeling over-protective, worried about health and ‘germophobic’
Pregnant group	‘Unlikely to allow people to touch my new-born.’
Parent group	‘It will make me more paranoid of others holding my baby. I am also more of a germaphobe.’
Independent and confident parenting	Spending more time with family; being more creative with activities at home; feeling more confident parenting independently as a result of having less support with child care, more resilient and more attentive to children's needs
Pregnant group	‘Having to trust my instincts in the absence of additional support, so think it will make me more confident and resilient.’
Parent group	‘I think it has made me feel more capable. I've had to learn to trust my own judgement and instincts.’
Not taking things for granted	Feeling more appreciative, socialising and going out more in the future
Pregnant group	‘It has really shown me not to take things for granted whereas before I may have not met up with family enough, etc. We will be making a great point to ensure that all family connections are high, and this is passed to baby.’
Parent group	‘I'll never take for granted having help with the baby from family again.’
Decrease in ability to parent	Feeling less confident as a parent and physically and mentally exhausted as a result of decreased support
Pregnant group	‘Scared to go out. Panic attacks.’
Parent group	‘I'm terrified to bring baby anywhere. I feel like a terrible parent.’
Worries about impact on mental health and well-being	Worries indirectly related to virus: worried about postnatal depression and mental health, impact of social isolation on child, and of passing own stress on to child
Pregnant group	‘I think I'll be quite anxious when the baby is born. My stress does impact on my parenting and I feel less patient.’
Parent group	‘I am a more anxious parent so may be more overprotective.’
Slowing down	Feeling less pressured to do things, becoming more reflective and patient
Pregnant group	‘Potentially move house and to a slower pace of life more family time orientated instead of making money to do expensive holidays to get that time.’
Parent group	‘It has helped me slow down and bond with my children.’

Overall, half of the themes reflected a negative impact on parenting (1, 4 and 5), whereas the other half reflected a positive impact (2, 3 and 6). For a full list of theme counts for expectant and new parents, please see Supplementary Appendix 4. For examples of male qualitative submissions only, please see Supplementary Appendix 5.

### Data synthesis

Two steps were used in the data synthesis. The first step of data synthesis was to interpret the data, matching the quantitative findings (i.e. factors associated with decrease in mood and confidence, irrespective of direction) with the themes that arose from the qualitative analysis of the three open-ended questions (see Supplementary Appendix 6). The second step of data synthesis was to identify barriers and facilitators to mood and confidence, based on the matching done in the first step (see Table 6).

**Table 6:** Barriers and facilitators to mood and parenting confidence

Mood		Parenting confidence	
Barriers	Facilitators	Barriers	Facilitators
Loss of physical contact	Support from others	Loss of physical contact	Support from others
Unreliable/inconsistent health information		Unreliable/inconsistent health information	Loss of child care
Difficulty in finding essentials		Difficulty in finding essentials	
Uncertainty during pregnancy		Uncertainty during pregnancy	
Decreased support (including loss of child care)		Decreased support (including loss of child care)	

Given that loss of child care was identified as both a barrier (i.e. decreased support) and facilitator, we ran an exploratory *post hoc* analysis to examine group differences between those who found it to be a barrier and those who found it to be a facilitator. The results indicated no demographic differences between groups. However, those who found loss of child care to be a barrier were more likely to be pregnant (odds ratio 5.09, *P* = 0.0019) and already have a child (odds ratio 7.05, *P* = 0.0001). This indicates that, although the uncertainty of being pregnant in combination with living with young children made loss of child care a challenge, this was not the case for first-time parents, for whom the experience of being entirely involved in their baby's care may have helped them feel more confident as parents (see Supplementary Appendix 7).

## Discussion

This study found that COVID-19 and associated restrictions during the most restrictive phase of lockdown had a detrimental impact on the well-being of expectant parents and those in early parenthood, resulting in a decrease in their mood and parenting confidence. This is consistent with recent findings of increased prenatal and postnatal anxiety,^19,20^ as well as increased mental distress in the wider population,^21^ during COVID-19. Our study also identified barriers and facilitators to well-being during this period. Although this was a predominantly female sample, we refer to parents generically, given that quantitative findings remained unchanged after removing male participants from analyses, and qualitative themes were found to be representative of both male and female perspectives.

### Barriers

Practical, social support and psychological difficulties were identified as barriers to both mood and parenting confidence. Practical difficulties included struggles to find essential items and reliable health information. Social support difficulties included not having a partner at appointments and full labour, losing child care and restricted access to healthcare support. This is in agreement with previous research showing that perceived low social support is a risk factor to postnatal depression in both parents,^22^ as well as to anxiety and antenatal and postnatal depression in women.^23,24^ Another important social barrier to mood and parenting confidence was the loss of physical contact (missing and/or avoiding it). Previous research has shown that touch may not only have a unique contribution to the formation of social bonds,^25^ conveying social support, but also act as a stress buffer, playing a critical regulatory role in the body's responses to acute life stressors.^26^ Another finding of this study was that being pregnant was associated with a decrease in mood during this period. Expectant parents reported that one of the hardest experiences was ‘not knowing’ the risks of COVID-19 for pregnant women, for the unborn and new-born child, the birth conditions during lockdown and what the future would entail. Uncertainty reduces our capacity to prepare for future threats and contributes to anxiety.^27^ For example, anxiety levels in pregnant women who reported abdominal pain and/or vaginal bleeding decreased over time when they were given a certain diagnosis, even if this was negative, whereas anxiety levels increased in those who received uncertain diagnoses.^28^ During pregnancy, expectant parents face several anxiety triggers: medical investigations into their own and their unborn child's health, waiting for results and sometimes uncertain diagnoses. The added uncertainty of risks brought about by COVID-19 may have negatively affected expectant parents’ mood more than that of parents, as expectant parents typically experience more uncertainty and anxiety when pregnant.

### Facilitators

The pandemic inevitably brought about difficulties in social support for expectant parents and those in early parenthood. However, support from others was identified as the greatest facilitator to mood and parenting confidence. Support was multidimensional, ranging from practical support with child care and pregnancy, to emotional and psychological support. The quantitative analysis revealed that high levels of current support from partner and friends, as well as increased family support, were associated with an increase in mood, and the latter was also associated with an increase in parenting confidence. However, the qualitative analysis revealed additional sources of support, mainly from healthcare professionals (e.g. midwives) and antenatal and postnatal groups. Perceived social support has been previously identified in the literature as a protective factor for parents’ emotional well-being.^29^ Interestingly, results from both quantitative and qualitative analyses indicated that loss of child care was experienced positively in relation to parenting confidence. Those who had to parent independently because of the loss of child care support resulting from COVID-19 restrictions reported feeling more confident in their ability to parent. However, loss of child care was also reported as one of the hardest aspects of lockdown. When looking at differences between groups, those who experienced loss of child care as a barrier were more likely to be pregnant, but not with their first child, and those who found it a facilitator were most likely to be first-time parents. These findings suggest that the uncertainty of being pregnant in combination with living with young children in the household presented a barrier to parents’ confidence. This is in line with recent research showing that parents with young children have higher levels of mental distress.^21^ Although there is evidence linking parenting confidence to children's later behaviour,^14^ there is little research focusing on which factors contribute to the development of parenting confidence.^30^ Our findings suggest that for first-time parents, not relying on child care had the positive effect of increasing their confidence in parenting. Qualitative submissions suggest that first-time parents found this experience helped them trust their instinct and judgement.

### The role of technology

The findings on participants’ views on technology are complex. Although the quantitative analysis revealed that the majority of participants found online interactions were less preferable than face-to-face interactions, the qualitative analysis showed that many participants named online resources and communication as being most helpful during lockdown. These findings suggest that technology can be a valuable tool to provide much needed practical, social, healthcare and psychological support in situations where these are not available face to face.

### Strengths and limitations

To the best of our knowledge, this is the first study to explore barriers and facilitators to mood and parenting confidence during pregnancy and early parenthood as a result of COVID-19 and its associated restrictions in the UK. Strengths of the study include, but are not limited to, a mixed-methods synthesis approach, integrating quantitative and qualitative data, the focus on the first and most restrictive phase of the lockdown, the representation of all locations within the UK and a range of incomes, and the anonymity of the data, which helped reduce response biases. Given the evidence of the disproportionate impact of COVID-19 on Black, Asian and minority ethnic groups,^31^ a major limitation was being unable to examine the role of ethnicity in our study. Further limitations were convenience sampling, which meant that findings were limited to the current sample, resulting in an underrepresentation of male participants and a possible underrepresentation of hard-to-reach populations.

### Conclusions and implications

This study and recent findings^19,20^ have demonstrated the negative impact of COVID-19 and associated restrictions on expectant parents and those in early parenthood. This is a cause for concern, given the association between parental well-being, parenting behaviour^6,7^ and child outcomes.^8,9^ Although worries increased, mood and confidence in one's ability to parent decreased, and this was found after 2 months in lockdown. These psychological difficulties are likely to increase as the socioeconomic impact of this period unfolds. Moreover, some restrictions continue and other restrictions are imminent as COVID-19 cases increase. This study also identified facilitators to mood and parenting confidence, which can help inform where resources should be focused. Despite the decline in social support, when this was received, it was much valued, confirming the importance of making partner, family, peer and healthcare support available, even if only remotely. It seems vital to investigate ways that technology can reach those in most need, and find better ways to provide support (e.g. technology that is user-friendly, valued and creatively used). For example, healthcare professionals could include partners in full labour, and face-to-face appointments with pregnant women and mothers through video or phone calls. However, pre-existing inequalities have been exacerbated with the pandemic,^32^ and special attention should be given to locations and groups of people who have little access to support and reliable health information, as well as access to technology. This will have benefits beyond the pandemic, given the potential of technology. Moreover, given the importance of touch for people's emotional well-being and for conveying social support, especially at a time of heightened stress, information on the importance of touch for adults and infants could be more widely considered with risks of transmission, to identify and clarify whether there are safe forms of physical contact. Finally, the inevitable uncertainty of the pandemic seems to affect expectant parents more severely, and so antenatal and postnatal healthcare and psychological support providers should be aware of this. Providing reassurance and guidance in managing anxiety during uncertain times could improve their well-being. We hope this research will be informative for individuals and organisations, who are now working with expectant parents and parents of infants, including, but not limited to, medical, mental health and social care professionals, to consider the psychological impact of this period, anticipate further repercussions and help offer best course of practice, given the particular limitations at any period of this pandemic.

## Data Availability

To gain access to the data supporting findings of this study, please contact the corresponding author, A.P., who will request approval from the UCL Research Ethics Committee.

## Supplementary material

For supplementary material accompanying this paper visit http://dx.doi.org/10.1192/bjo.2021.925. click here to view supplementary material
