# Supplementary material for: Barriers and facilitators to mood and confidence in pregnancy and early parenthood during COVID-19 in the UK: mixed-methods synthesis survey
Source: BJPsych Open. 2021 Jun 1;7(4):e107. doi: 10.1192/bjo.2021.925 (PMC8167260; doi:10.1192/bjo.2021.925)
Supplement: Supplementary file 1 [file S205647242100925Xsup001.zip › Supplement_7._Loss_of_childcare.docx]

***Supplement 7. Fisher’s exact test and counts for loss of childcare***

|  | **First baby/pregnancy** | **Not first baby/pregnancy** |
| --- | --- | --- |
| Facilitator | 20 | 11 |
| Barrier | 9 | 36 |

Table 7.1: Previous parent experience counts for loss of childcare as a barrier or facilitator

*Fisher’s exact test* OR = 7·05, 95% CI 2·31 – 23.45, *p* = 0·0001

|  | **Parent** | **Expectant parent (pregnant)** |
| --- | --- | --- |
| Facilitator | 25 | 6 |
| Barrier | 20 | 25 |

Table 7.2: Parent and expectant parent counts for loss of childcare as a barrier or facilitator

*Fisher’s exact test* OR = 5.09, 95% CI 1·63 – 18·24, *p* = 0·0020
