# Supplementary material for: Barriers and facilitators to mood and confidence in pregnancy and early parenthood during COVID-19 in the UK: mixed-methods synthesis survey
Source: BJPsych Open. 2021 Jun 1;7(4):e107. doi: 10.1192/bjo.2021.925 (PMC8167260; doi:10.1192/bjo.2021.925)
Supplement: Supplementary file 1 [file S205647242100925Xsup001.zip › Supplement_6._Data_synthesis.docx]

*Supplement 6. Data synthesis for parental mood and confidence*

| **Quantitative Findings** | **Qualitative Themes** |
| --- | --- |
| Missing physical contact | Loss |
| Avoiding physical contact | Loss |
| Not finding reliable health information | Unreliable/inconsistent information |
| Difficulty in finding essentials | Practical difficulties |
| Being pregnant | Uncertainty during pregnancy |
| High current partner support | Support from others/Decreased support |
| High current friends’ support | Support from others/Decreased support |
| Increase in family support | Support from others/Decreased support |

Table 6.1: Matching quantitative findings for mood with qualitative themes

| **Quantitative Findings** | **Qualitative Themes** |
| --- | --- |
| Missing physical contact | Loss |
| Increase in searching for reliable health information | Unreliable/inconsistent information |
| Difficulty in finding essentials | Practical difficulties |
| Losing childcare | Independent and confident parenting |
| Increase in family support | Support from others/decreased support |
| Being pregnant | Uncertainty during pregnancy |

Table 6.2: Matching quantitative findings for confidence in parenting with qualitative themes
