# Supplementary material for: Barriers and facilitators to mood and confidence in pregnancy and early parenthood during COVID-19 in the UK: mixed-methods synthesis survey
Source: BJPsych Open. 2021 Jun 1;7(4):e107. doi: 10.1192/bjo.2021.925 (PMC8167260; doi:10.1192/bjo.2021.925)
Supplement: Supplementary file 1 [file S205647242100925Xsup001.zip › Supplement_5._Qualitative_male_only.docx]

| No | ***Theme*** | ***Examples of submissions assigned to each theme*** |
| --- | --- | --- |
|  | **“Hardest”** |  |
| 1 | Decreased support | Healthcare*:“Partner suffering very bad morning sickness and not being able to visit midwife”*  Family*: “Not having support of family to give us a break”* |
| 2 | Loss | *“Not being able to introduce our newborn son to his grandparents”* |
| 3 | Worry | *“I am worried about contracting something when getting shopping that I then pass to my baby”* |
| 4 | Having to be at home | *“Not been able to take them places”* |
| 5 | Uncertainty during pregnancy | *“Not knowing if I'll be allowed to be present at the birth”* |
| 6 | Practical difficulties | *“Wife being stuck inside and having to be the only person who can go shopping”* |
| 7 | Unreliable/inconsistent information | *“Everything we were told before covid-19 was about how important it was to start treatment as early as possible and now it is just cancelled.”* |
|  | **“Most helpful”** |  |
| 1 | Support from others | *“Good telephone support from medical staff”* |
| 2 | More time | *“Being able to work from home and spend more time with family and partner”* |
| 3 | Technology | *“Video calling platforms are useful”* |
| 4 | No pressure | *“Not having to travel - and not expected to do as much work”* |
| 5 | Nothing | *“Nothing”* |
|  | **“Influence on future parenting/parenting”** |  |
| 1 | Hygiene and restrictions of socialising | *“More cautious in regards to hygiene and health”* |
| 2 | Independent and confident parenting | *“Being more comfortable to be independent”* |
| 6 | Slowing down | *“Further prioritise my family over work, considering condensing work hours into fewer days”* |

***Supplement 5. Qualitative submissions of male respondents only***
