# Supplementary material for: Barriers and facilitators to mood and confidence in pregnancy and early parenthood during COVID-19 in the UK: mixed-methods synthesis survey
Source: BJPsych Open. 2021 Jun 1;7(4):e107. doi: 10.1192/bjo.2021.925 (PMC8167260; doi:10.1192/bjo.2021.925)
Supplement: Supplementary file 1 [file S205647242100925Xsup001.zip › Supplement_4._Counts_for_themes.docx]

***Supplement 4. Counts for qualitative themes***

| **Theme** | **Count** |
| --- | --- |
| **1. Decreased support**  Pregnant group  Parent group       Both groups | 252  181  **433** |
| **2. Loss**  Pregnant group  Parent group  Both groups | 150  132  **282** |
| **3. Worry**  Pregnant group  Parent group  Both groups | 79  59  **138** |
| **4. Having to be at home**  Pregnant group  Parent group  Both groups | 80  51  **131** |
| **5. Uncertainty during pregnancy**  Pregnant group  Parent group  Both groups | 45  3  **48** |
| **6. Practical difficulties**  Pregnant group  Parent group  Both groups | 19  5  **24** |
| **7. Unreliable information**  Pregnant group  Parent group  Both groups | 10  1  **11** |

Table 4.1: Theme counts for what was “hardest” during lockdown

| **Theme** | **Count** |
| --- | --- |
| **1. Support from others**  Pregnant group  Parent group       Both groups | 137  67  **204** |
| **2. More time**  Pregnant group  Parent group  Both groups | 83  102  **185** |
| **3. Technology**  Pregnant group  Parent group  Both groups | **59**  **44**  **103** |
| **4. No pressure**  Pregnant group  Parent group  Both groups | 45  38  **83** |
| **5. Nothing**  Pregnant group  Parent group  Both groups | 42  11  **53** |

Table 4.2: Theme counts for what was “most helpful” during lockdown

| **Theme** | **Count** |
| --- | --- |
| **1. Hygiene and restrictions of socialising**  Pregnant group  Parent group       Both groups | 117  52  **169** |
| **2. Independent and confident parenting**  Pregnant group  Parent group  Both groups | 30  45  **75** |
| **3. Not taking things for granted**  Pregnant group  Parent group  Both groups | 27  21  **48** |
| **4. Decrease in ability to parent**  Pregnant group  Parent group  Both groups | 31  10  **41** |
| **5. Worries about impact on mental health and well-being**  Pregnant group  Parent group  Both groups | 13  9  **22** |
| **6. Slowing down**  Pregnant group  Parent group  Both groups | 12  8  **20** |

Table 4.3: Theme counts for “influence on future parenting/parenting”
