# Supplementary material for: Barriers and facilitators to mood and confidence in pregnancy and early parenthood during COVID-19 in the UK: mixed-methods synthesis survey
Source: BJPsych Open. 2021 Jun 1;7(4):e107. doi: 10.1192/bjo.2021.925 (PMC8167260; doi:10.1192/bjo.2021.925)
Supplement: Supplementary file 1 [file S205647242100925Xsup001.zip › Supplement_3._Model_summaries.docx]

***Supplement 3. Model summaries***

Model summaries for change in mood since COVID-19 are presented below. Mood prior to COVID-19 was highly associated with mood change in all models since it is obviously correlated (e.g. your mood cannot decrease if your prior mood is at the lowest point of the measure). Of the demographic covariates, positive associations with income and accommodation and negative associations with being pregnant were detected in some models.

| **Coefficients** | **Estimate** | **Std error** | **T value** | ***p*** |
| --- | --- | --- | --- | --- |
| Missing physical contact | -0·15 | 0·03 | -4·40 | < 0·0001 |
| Avoiding physical contact | -0·22 | 0·09 | -2·39 | 0·017 |
| Income | 0·04 | 0·02 | 1·71 | 0·088 |
| accommodation | 0·07 | 0·04 | 1·52 | 0·13 |
| Pregnant | -0·14 | 0·07 | -1·93 | 0·054 |
| Mood prior to COVID-19 | -0·75 | 0·04 | -16·81 | < 0·0001 |

Table 3.1: Model of change in mood since COVID-19 and physical contact

*Overall model fit* *F*(6, 576) = 55·94 , *p* < 0·0001, *R^2^* = 0·37

| **Coefficients** | **Estimate** | **Std error** | **T value** | ***p*** |
| --- | --- | --- | --- | --- |
| Frequency of searching for reliable health information | -0·12 | 0·07 | -1·76 | 0·079 |
| Not finding reliable health information | -0·30 | 0·08 | -3·92 | < 0·0001 |
| No previous parenting experience | -0·10 | 0·07 | -1·30 | 0·19 |
| Income | 0·05 | 0·02 | 1·97 | 0·050 |
| Mood prior to COVID-19 | -0·78 | 0·05 | -16·58 | < 0·0001 |

Table 3.2: Model of change in mood since COVID-19 and use of media

*Overall model fit F*(5, 533) = 57·92, *p* < 0·0001, *R^2^* = 0·35

| **Coefficients** | **Estimate** | **Std error** | **T value** | ***p*** |
| --- | --- | --- | --- | --- |
| Total stress events experienced | -0·06 | 0·04 | -1·56 | 0·12 |
| Difficulty finding essentials | -0·27 | 0·08 | -3·25 | 0·0012 |
| Income | 0·05 | 0·02 | 2·40 | 0·017 |
| Mood prior to COVID-19 | -0·79 | 0·04 | -17·93 | < 0·0001 |

Table 3.3: Model of change in mood since C-19 and stress events

*Overall model fit F*(4, 585) = 83·79, *p* < 0·0001, *R^2^* = 0·36

| **Coefficients** | **Estimate** | **Std error** | **T value** | ***p*** |
| --- | --- | --- | --- | --- |
| Support from partner since COVID-19 | 0·16 | 0·06 | 2·66 | 0·0076 |
| Support from friends since COVID-19 | 0·16 | 0·06 | 2·73 | 0·0065 |
| Change in support from friends since COVID-19 | -0·09 | 0·06 | -1·45 | 0·15 |
| Support from family since COVID-19 | 0·23 | 0·05 | 4·24 | < 0·0001 |
| Income | 0·05 | 0·02 | 2·22 | 0·027 |
| Accommodation | 0·08 | 0·04 | 1·84 | 0·067 |
| Pregnant | -0·21 | 0·07 | -2·88 | 0·0041 |
| Mood prior to COVID-19 | -0·83 | 0·05 | -17·31 | < 0·0001 |

Table 3.4: Model of change in mood since COVID-19 and support

*Overall model fit F*(8, 553) = 47·00, *p* < 0·0001, *R^2^* = 0·40
