# Supplementary material for: Barriers and facilitators to mood and confidence in pregnancy and early parenthood during COVID-19 in the UK: mixed-methods synthesis survey
Source: BJPsych Open. 2021 Jun 1;7(4):e107. doi: 10.1192/bjo.2021.925 (PMC8167260; doi:10.1192/bjo.2021.925)
Supplement: Supplementary file 1 [file S205647242100925Xsup001.zip › Supplement_2._Quantitative_method.docx]

## Supplement 2. Details of quantitative analysis

### Quantitative study variables.

Participant characteristics including gender, age, expectant/new parent status, previous parent experience, work situation, income, accommodation, and location in the UK were included as candidate covariates for variable selection. Details of participant characteristics and a summary of study highlights are presented in the manuscript.

### Statistical modelling.

Variables of interest were grouped into 4 different themes:

1. Support – models for this theme included the following variables: current support from partners, friends and family, the change in support from partners, friends and family since COVID-19. Change in support is calculated by scoring the ordered categories in the current and prior support variables from 1 to 4, with 1 indicating no support and 4 indicating highly supported, and deducting prior from current support, giving a number between -3 to 3, with negative values indicating a decrease in support.
2. Physical contact – models for this theme included the following variables: worries about having physical contact with others in the household, avoiding contact with others in the household, and missing physical contact more generally.
3. Use of media – models for this theme included the following variables: preference of connecting with others online, frequency of looking at social media, frequency of looking at news, frequency of searching for reliable health information, and finding a reliable source of health information.
4. Stress events – models for this theme included a cumulative number of stress events experienced (out of a total of 11) and the four most frequently reported stress events: having to isolate from friends / family members, having to attend hospital / GP appointments on their own, having difficulty accessing medicine, groceries or other essential items, and losing access to childcare.

Two outcomes were examined: change in mood since COVID-19 and impact of COVID-19 on confidence in parenting. Change in mood is calculated by scoring the ordered categories in the current and prior mood variables from 1 to 5, with 1 indicating very sad and 5 indicating very happy, and deducting prior from current mood, giving a number between -4 to 4, with negative values indicating a decrease in mood. Mood prior to COVID-19 was included as a covariate when modelling change in mood as it is correlated and therefore needs to be controlled for.

Models were fit using linear regression or proportional odds modelling depending on whether the outcome was continuous or ordinal respectively.

For each outcome, a model was fit using just covariates, to identify possible associations with demographic variables.

Models were then fit including a single themed group of factors, controlling for the covariates. Variable selection used stepwise methods, minimizing Akaike information criteria (AIC). As this analysis was based on a convenience sample and was exploratory in nature, we did not use any post hoc correction as the additional rigor is disproportionate.

### Change of mood since COVID-19.

All models of mood changes were multiple linear regression models, with mood change coded as a scale variable taking values between -4 and 4. This was calculated by scoring both previous and current mood from 1 to 5 (with 1 indicating “Very sad” and 5 indicating “Very happy”), and deducting previous mood from current mood.

Effect sizes were interpreted as the average change in mood associated with changes in the appropriate variable of interest. A positive effect means an increase in the average change in mood as the variable increases. A negative effect is a decrease in the average change in mood as the variable increases. For example, a change of mood of one level could indicate an increase from 1 to 2 (a stronger increase in happiness) or from -2 to -1 (remaining sad, but less so).

Only 24 responses indicated an improvement in mood since COVID-19, so in general, positive associations most often relate to participants remaining sad, but less so.

All models of mood changes included the happiness before COVID-19 as a covariate because this was highly correlated with mood change (for example, consider that your mood cannot worsen if it is already poor).

### Confidence in parenting since COVID-19.

All models of confidence in parenting were proportional odds models and effect sizes indicated the change in odds associated with the variable of interest. In models where previous parenting experience was a variable selected in the model, the proportional odds assumption was violated (as determined using the Brant test). Partial proportional odds models were fit when this occurred. Since we were seeking potential associations and not inferring effects, and the conclusions did not change, we reported the proportional odds conclusions, since they were robust to the violation.
