# Supplementary material for: Barriers and facilitators to mood and confidence in pregnancy and early parenthood during COVID-19 in the UK: mixed-methods synthesis survey
Source: BJPsych Open. 2021 Jun 1;7(4):e107. doi: 10.1192/bjo.2021.925 (PMC8167260; doi:10.1192/bjo.2021.925)
Supplement: Supplementary file 1 [file S205647242100925Xsup001.zip › Supplement_1._COVID-19_pregnancy_and_new_parent_survey.docx]

***Supplement 1. Participant information sheet and survey***

**Information sheet**

**Information sheet for participants**

UCL Research Ethics Committee Approval ID Number: 7683*/*003

**Learning about pregnancy and early parenting experiences during Coronavirus *(*COVID-19)**

Research Team:

Dr Alejandra Perez ([alejandra.perez@annafreud.org](mailto:alejandra.perez@annafreud.org))

Dr Elena Panagiotopoulou ([elena.panagiotopoulou@annafreud.org](mailto:elena.panagiotopoulou@annafreud.org))

Ruth Roberts ([ruth.roberts@annafreud.org](mailto:ruth.roberts@annafreud.org))

Address: Anna Freud Centre, Kantor Centre of Excellence, 4-8 Rodney Street, London, N1 9JH

Why are we carrying out this study? The research team from the Anna Freud National Centre for Children and Families (AFNCCF) are seeking to learn about how pregnancy and early parenting experiences are uniquely affected by changes and restrictions due to Coronavirus (COVID-19). We are asking people who are currently pregnant or their partner is pregnant or who have become a parent in the last 6 months to take part in this study*. This study is open to men and women who are residents of the UK and who are 18 years of age or older. W*hat will happen if I take part? If you decide to take part, you will be asked to complete a short online survey asking about different experiences you may have had during pregnancy or early parenthood during COVID-19. The survey should not take longer than 10 minutes to complete. We will not ask you to provide any individually identifying information (such as your name or email address).

Do I have to take part? There is no obligation for you to take part and your decision will not disadvantage you in any way. Importantly, even if you do decide to take part, you can stop at any time without needing to give a reason. If you exit the survey without clicking on the submit button, we will understand that you would like to withdraw your participation and your data will be securely deleted. What are the possible disadvantages to taking part? We do not anticipate any risk of taking part in this study, howe*v*er, sometimes it can feel difficult to share your experiences. You do not have to share anything that makes you uncomfortable and you are free to skip any questions that you do not wish to answer. You are free to exit the survey at any time. A list of mental health resources will be provided at the end of the survey should you wish to access confidential support. What are the possible benefits of taking part? Your participation will help us learn about how pregnancy and early parenthood is experienced during times of change and uncertainty and understand how we can best support new parents and those who were expecting a baby during COVID-19. What will happen to the information you collect?

The information may be used for scientific presentations and publications. *Y*our information will be kept private and confidential.

The information you provide will be stored securely for up to a maximum of 10 years. Due to the anonymous nature of the questionnaire, it will not be possible to withdraw your data after you click the submit button. Ask questions! If you have any questions, please feel free to email the research team at the details above. If you decide to take part and feel, either during or after your participation, that you have been treated unfairly or would like to raise a complaint you can contact the study's Principal Investigator, Dr Alejandra Perez (alejandra.perez@annafreud.org). Should you feel that your complaint has not been handled to your satisfaction, please contact the Chair of the UCL Research Ethics Committee (ethics@ucl.ac.uk).

*Data Protection Privacy Notice: The data controller for this project will be University College London (UCL). The UCL Data Protection Office provides oversight of UCL activities involving the processing of personal data, and UCL's Data Protection Officer can be contacted at data-protection@ucl.ac.uk. If you are concerned about how your personal data is being processed, please contact UCL in the first instance at data protection@ucl.ac.uk. If you remain unsatisfied, you may wish to contact the Information Commissioner's Office (ICO).*

https*://u*clpsych.eu.qualtrics.com*/*Q/EditSection*/*Block*s/*Aja*x/*GetSurveyPrintPreview?ContextSurveyID=SV_1KVJAKdmWmgiyd7&ContextLibrar...

*1/*10

21*/09/2*020

Qualtrics Survey Software *Contact details, and details of data subject rights, are available on the ICO website at: https://ico.org.uk/for organisations/data-protection-reform/overview-of-the-gdpr/individuals-rights/*

*Thank you for reading this information sheet*.

I have read the information sheet and I consent to take part

I do NOT consent to take part

**Please tell us *y*our gender**

**Male**

**Female**

If you do not identify as male or female, please specify below

**Are you:**

Pregnant (first trime**ster)** Pregnant (second trimester) Pregnant (third trimester) Parent of a baby (up to 1 month) Parent of a baby (up to 3 months) **Parent** of a baby (up to 6 months)

**Is this your first pregnancy?**

Yes

**No**

**Is this your first child?**

Yes

**No**

**Are you or your partner:**

Currently working in the NHS or a**s a care worker**

**htt**ps*://*uclpsych.eu.qualtrics.co*m/*Q/EditSection/Blocks/Aja*x/*GetSurveyPrintPreview?ContextSurveyID=SV_1K*V*JAKdmWmgiyd7&ContextLibrar...

2*/1*0

2*1/*0*9/2*020

Qualtric**s Survey Software** Currently going to work and having contact with the public (but not NHS or **care worker) None of the above**

**The next section will be about your feelings and experiences BEFORE COVID-19**

**Before COVID-19, how supported did you feel by your:**

Not supported

Minimally supported

Highly **supported**

**Supported**

**Not applicable**

**Partner**

**Friends**

**Family**

**Before COVID-19, how would you rate your general mood:**

***V*ery sad**

**Sad**

Neutral – neither happy or sad

Happy Very happy

The next section will be about your feelin**gs and experiences SINCE COVID-19**

Since **COVID-19 have you experienced any of the following? (tick all that apply)**

Lost your job Been furloughed

**Had to move acco**mmodation Had to return from a different country Had to attend hospital / GP appointments on your own **Lost access t**o childcare Had to isolate from friends */* family members Had difficulty **accessing m**edicine, gr**oceries or other essential items** Contracted COVID-19 **Had a close frien**d or family m**ember become seriou**sly ill from COVID-19

**Experienced bereavement related to CO***V*ID-19

None of the **above**

https*://u*clpsych.eu.qualtrics.com*/*Q/EditSection*/***Block*s/*Aja*x/*GetSurveyPrintPreview?ContextSurveyID=SV_1KVJA**KdmWmgiyd7&ContextLibrar...

*3/*10

2*1/*09*/*2020

**Qualtrics Survey Software**

**Since COVID-19 have you experienced any of the following? (tick all that apply)**

Lost your job Been furloughed

**Had to move accommodation Had to return from a different country** Had to give birth on your own Had to attend hospital/GP appointments on your own **Lost access t**o childcare

Had to isolate from friends*/*family me**mbers**

Had to isolate from your n**ewborn** Had difficulty accessing medicine, groceries or other essential items Contracted COVID-19 Had a close friend or family became seriously ill with COVID-19 **Experienced bereavement related t**o COVID-19

None of the a**bove**

Sinc**e COVID-19, how supported do you feel by your:**

Not **supported**

Minimally supported

Highly supported

Highly

**Not** applicable

applicable

Supported

**Partner**

**Friends**

**Family**

Since **COVID-19, have you found connecting online with others:**

**More prefera**ble than meeting them **face-to-face**

**The same as meeting them face-to-face**

**Less preferable than meetin**g them face-to-face

Not applicable - Yo**u are not connectin**g with others online

Since C**OVID-19, have you found yourself:**

**About the**

**same**

**Less often**

More often

Not applicable

**Looking at news Looking at social media Searching for reliable health information**

https*://u*clpsych.eu.qualtrics.com*/*Q/EditSection*/***Block*s/*Aja*x/*GetSurveyPrintPreview?ContextSurveyID=SV_1KVJA**KdmWmgiyd7&ContextLibrar...

4*/*10

2*1/*09*/*2020

**Qualtrics Survey Software**

**Have you found a reliable source of health information?**

No

**Yes - please indicate source**

Since **COVID-19, have you found yourself feeling:**

*M*ore worried about the health of your unborn baby

**The same amount of worry as before CO**VID-19 about the health of your unborn baby **Less w**orried about the health of your unborn baby

Sinc**e COVID-19, have you found yourself feeling:** More worried about the health of your baby **The same amount of worry as befo**re COVID-19 about the health of your baby **Less worried** about the health of your baby

Since **COVID-19, have you found yourself feeling:**

More worried about your own health

**The same amount of worry as before CO**VID-19 about your own health

**Less worried about your own health**

Since COVID-19**, how would you rate your general mood:**

**Very sad**

**Sad**

Neutral – neither happy or sad Happy *V*ery happy

Since **COVID-19, have you been worried about having physical contact with others in your household?**

**Yes**

No

Since **CO*V*ID-19, have *y*ou found yourself avoiding physical contact with others in your household?**

https*://u*clpsych.eu.qualtrics.com*/*Q/EditSection*/***Block*s/*Aja*x/*GetSurveyPrintPreview?ContextSurveyID=SV_1KVJA**KdmWmgiyd7&ContextLibrar...

5*/*10

21*/09/2*020

Qualtrics Sur**vey Software**

**Yes**

**No**

Since **COVID-19, how much do you miss physical contact with others more generally?**

A great deal

A lot **A moderate amount**

A little

Not at all

Not applicable

Since **COVID-19, have you been worried about having physical contact with your baby?**

**Yes**

**No**

**Since COVID-19, have you found yourself avoiding physical contact with your baby?**

**Yes No**

Since **COVID-19, have you found yourself restricting your baby's physical contact with others in your household?**

**Yes**

**No**

Since **COVID-19, have you felt:**

**More aware of your baby's movements As aware as before of *y*our baby's movements Less aware of your baby's movements**

Sinc**e COVID-19, have you felt:**

**More aware of** your baby's needs (for example, need for attention, to be held, fed, changed, soothed) **As aware as before of your baby's needs Less aware** of your baby'**s needs**

https*://u*clpsych.eu.qualtrics.com*/*Q/EditSection*/***Block*s/*Aja*x/*GetSurveyPrintPreview?ContextSurveyID=SV_1KVJA**KdmWmgiyd7&ContextLibrar...

6*/*10

2*1/*09*/*2020

Qualtrics Sur**vey Software**

**Since COVID-19, have you felt:**

*M*ore worried about going to hospital*/*medical appointments or home **visits** Neither more or less worried about going to hospital/ medical appointments or home visits **Less worried** about going to hospital*/* medical appointments or home visits

**What has been the hardest part of being pregnant during COVID-19?**

**What has been the hardest part of being a parent of a new baby during COVID-19?**

**What have *y*ou found to be most helpful while being pregnant during COVID-19?**

**What have you found to be most helpful while being a parent of a new baby during COVID-19?**

Since **COVID-19, do you feel:**

More confident in yourself as an expectant parent **The same as before in terms** of confidence in yourse**lf as an expectant parent**

**Less c**onfident in yourself as a**n expectant parent**

Sinc**e COVID-19, do you feel:**

More confident in yoursel**f as a parent http**s*://*uclpsych.eu.qualtrics.co*m/Q/*EditSection/Blo**cks/Aja*x/*GetSurveyPrintPreview?ContextSurveyID=SV_**1KVJAKdmWmgiyd7&ContextLibrar..*.*

*7/1*0

2*1/*09*/*2020

Qualtrics Surv**ey Software**

**The same as before in t**erms of your confidenc**e as a parent**

**Less** confident in yourse**lf as a parent**

**Do you think this experience of COVID-19 will influence your future parenting?**

**No**

**Yes (please tell us how below)**

**Do you think this experience of COVID**-19 will i**nfluence your parenting?**

**No**

**Yes (please tell us how below)**

**Where in the UK do you live? (city */* town)**

**What type of accommodation do you currently live in?**

Flat with no view

Flat with vie*w /* balcony

Flat with garden */* **terrace** */* outside space House with garden */* **terrace */* outside space**

**Please tell us your age:**

**18-24 years**

**25-34 years 35-44 years 45+**

**Please tell us your ethnicity:** https*://u*clpsych.eu.qualtrics.com*/*Q/EditSection*/***Block*s/*Aja*x/*GetSurveyPrintPreview?ContextSurveyID=SV_1KVJA**KdmWmgiyd7&ContextLibrar...

8*/*10

2*1/*09*/*2020

**Qualtrics Survey Software**

White (including British, Irish, any other White background) Mixed (including White & Black Caribbean, White & Black African, White & Asian, any other mi**xed** background)

Black or Black British (Caribbean, African, any other Black background) Asian or Asian British (Indian, Pakistani, Bangladeshi, any other Asian background) Other ethnic groups (Chinese, any other ethnic group)

Prefer not to say

**Please tell us your total household income:**

Up to £20,999 £21,000 - £40,000 £41,000 - £60,000 £61,000 - £80,000 £81,000 - £100,000 More than £100,000

**Submit my data:**

Yes

No

Thank you for taking part in this survey.

For confidential support and advice for your well-bein**g here are some relevant resources:**

*World Health Organisation*

**https*://www.*who.int*/*news-room*/*campaigns*/*connecting-the-world-to-combatcoronavirus*/*healthyathome*/*healthyathome--mental-health**

*Mind*

https*://www.*mind.org.uk*/*

**S*amaritans***

**https*://w*w*w.*samaritans.org*/*how-we-can-help/support-and**-information/

*NHS mental health helplines*

https*://u*clpsych.eu.qualtrics.com*/*Q/EditSection*/***Block*s/*Aja*x/*GetSurveyPrintPreview?ContextSurveyID=SV_1KVJA**KdmWmgiyd7&ContextLibrar...

*9/*10

2*1/*09*/*2020

2. pap2020.

Qualtrics Surv**ey Software**

te solutions Suray Sonwen

**https*://w*w*w*.nhs.uk*/*conditions*/*stress-anxiety-depression*/*mental-hea**lth-helplines*/*

**Powered by Qualtrics**

https*://u*clpsych.eu.qualtrics.com*/*Q/EditSection*/***Block*s/*Aja*x/*GetSurveyPrintPreview?ContextSurveyID=SV_1KVJA**KdmWmgiyd7&ContextLibra...

10*/*10
